# Supplementary material for: Synthetic lethal interaction between WEE1 and PKMYT1 is a target for multiple low-dose treatment of high-grade serous ovarian carcinoma
Source: NAR Cancer. 2023 Jun 13;5(3):zcad029. doi: 10.1093/narcan/zcad029 (PMC10262308; doi:10.1093/narcan/zcad029)
Supplement: zcad029_Supplemental_File [file zcad029_supplemental_file.pdf]

Supplementary Figure 1. - Combined inhibition of WEE1 and PKMYT1 synergize in killing of cancer cells

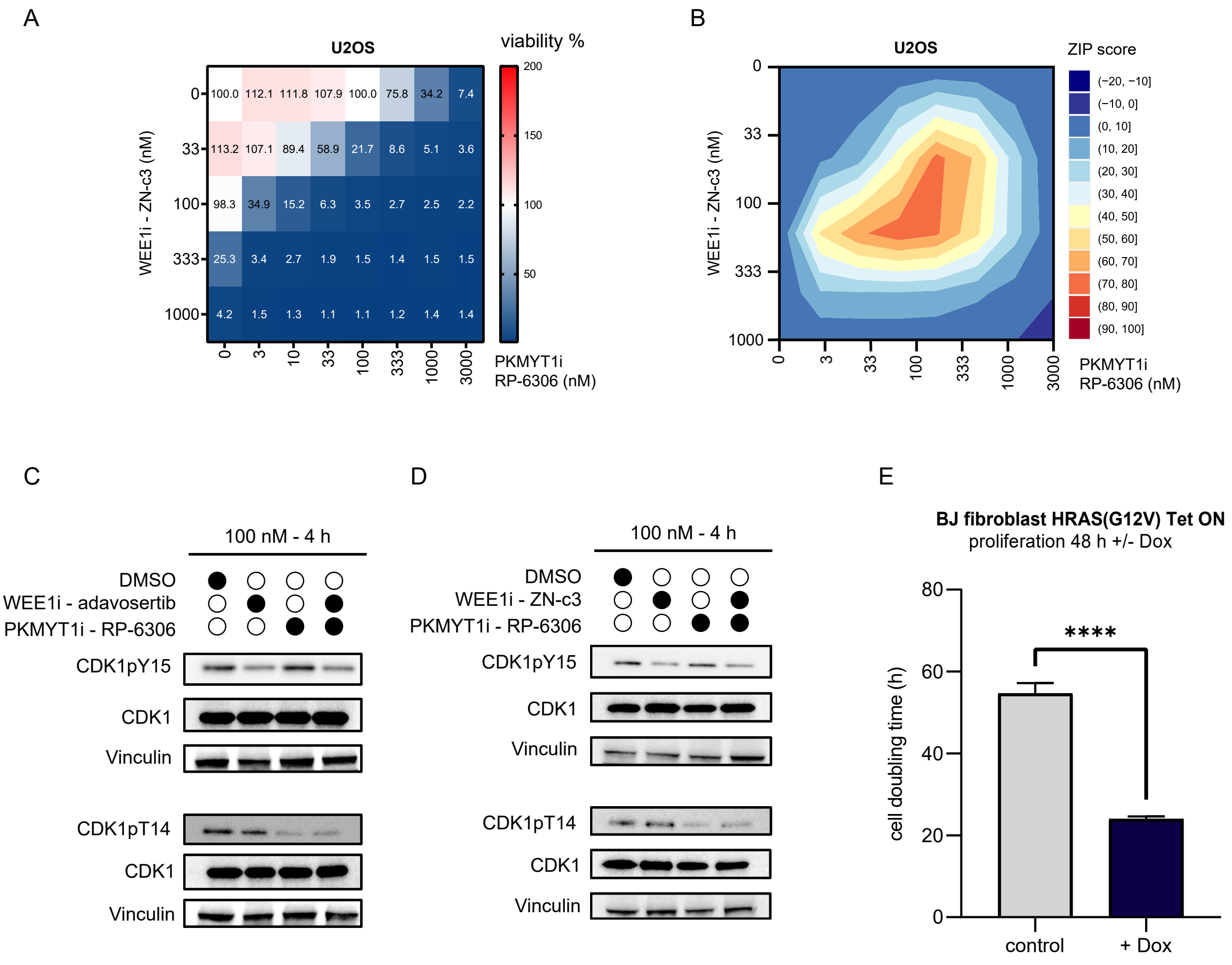

Supplementary Figure 2. - Combined inhibition of WEE1 and PKMYT1 synergize in killing of cancer cells

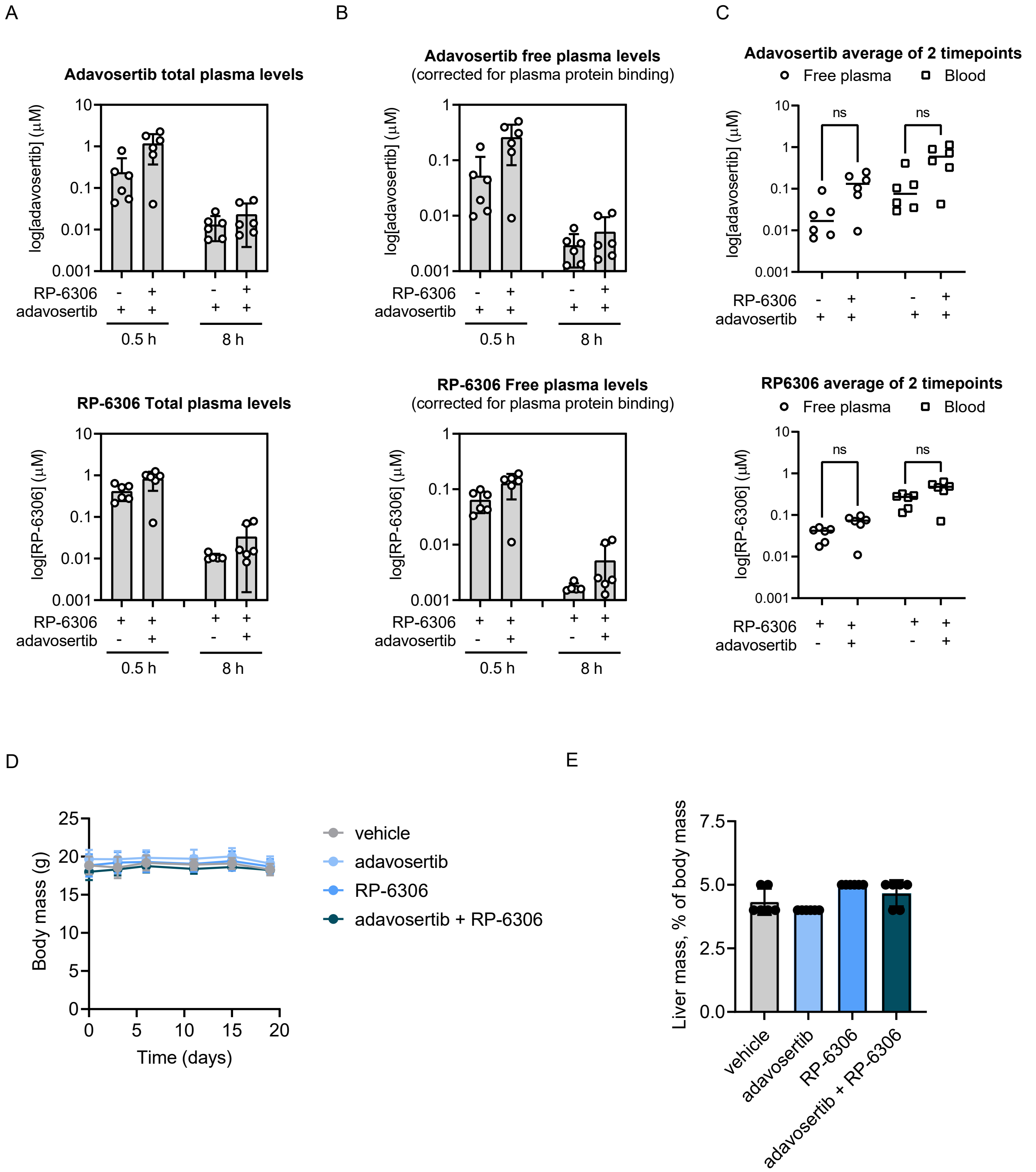

Supplementary Figure 3. - WEE1 and PKMYT1 co-inhibition exacerbates replication stress and triggers replication catastrophe

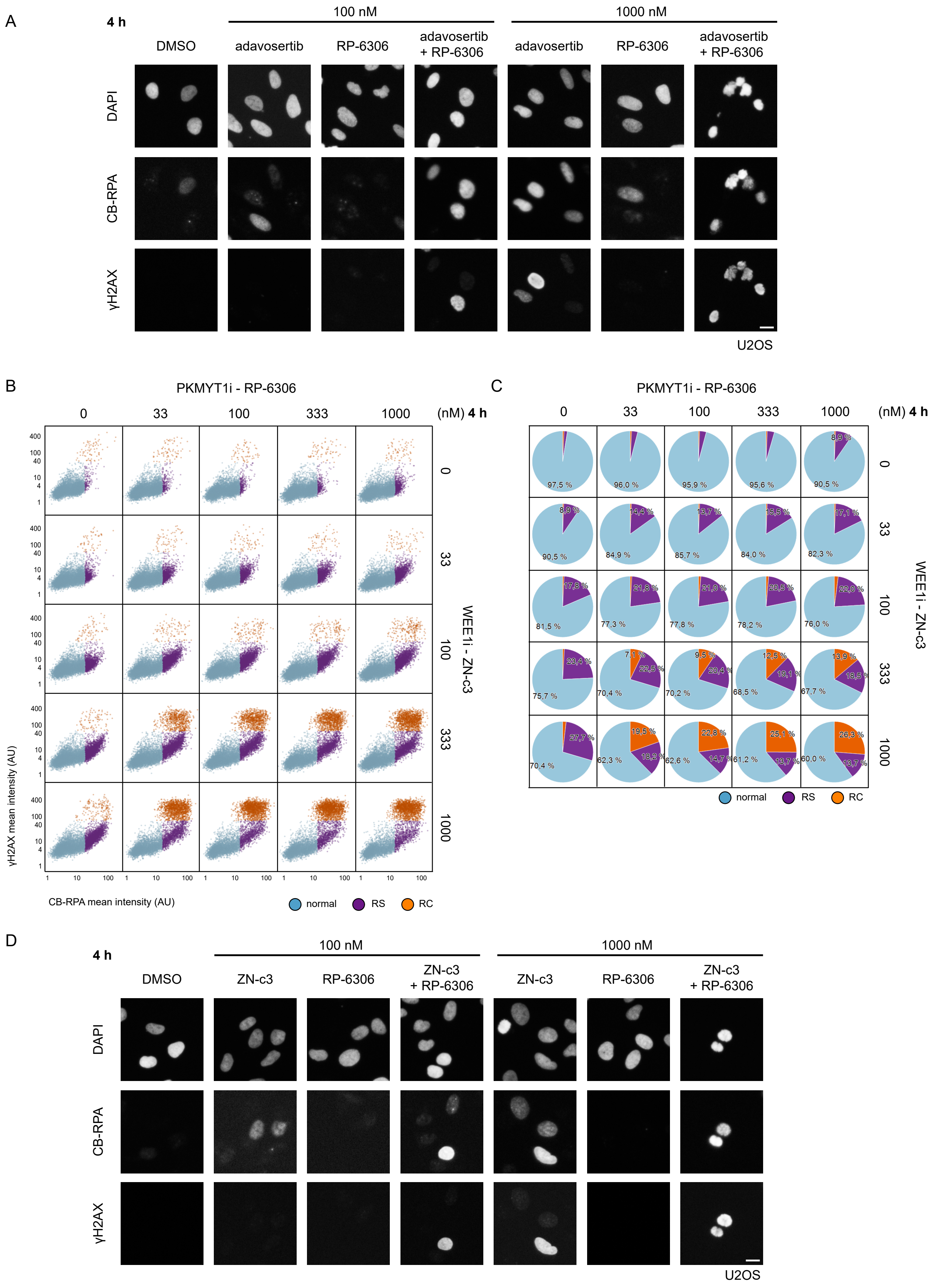

Supplementary Figure 4. - WEE1 and PKMYT1 gene expression in ovarian cancer and gene expression of driver oncogenes in panel of HGSC cell lines

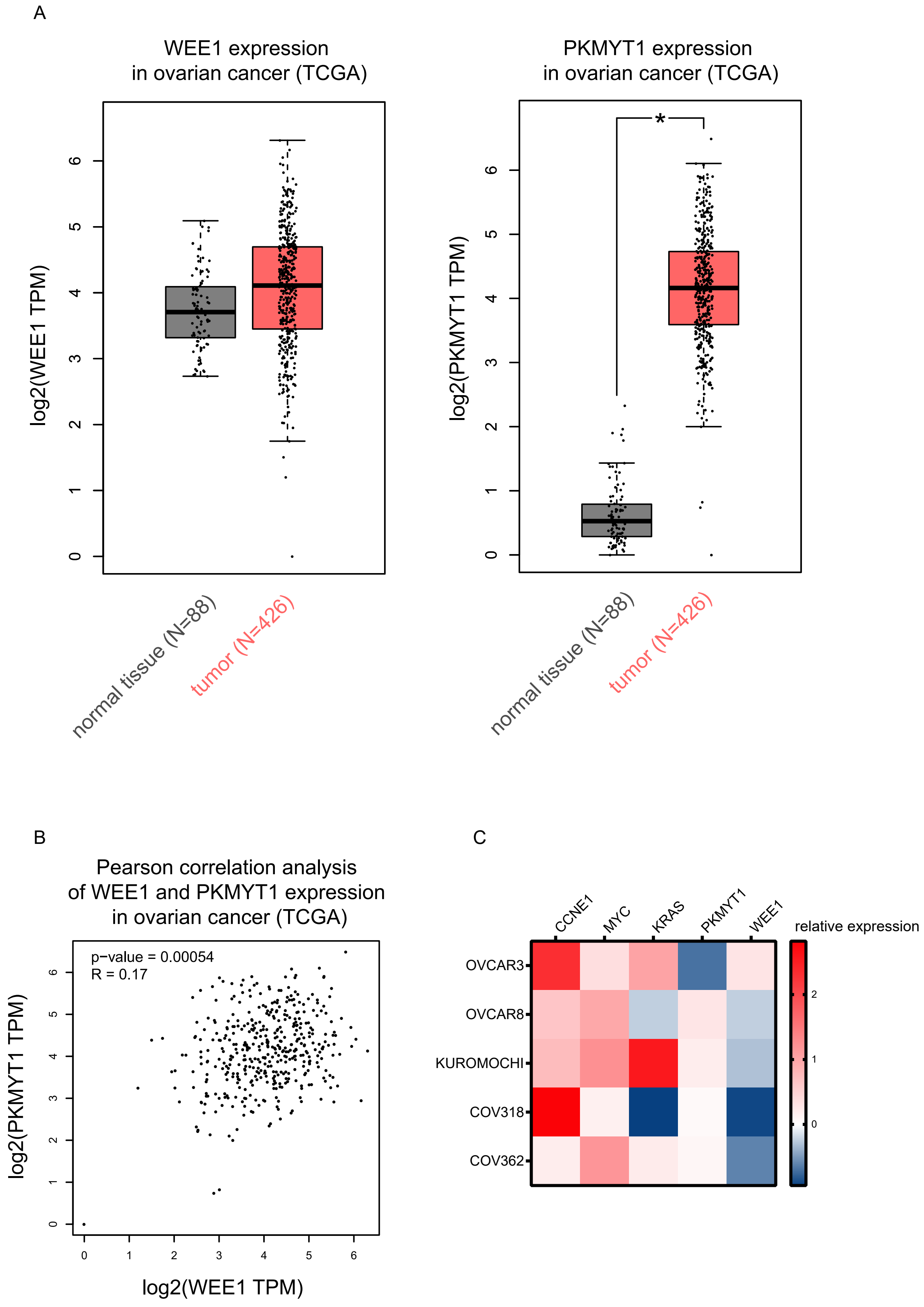

Supplementary Figure 5. - WEE1 and PKMYT1 co-inhibition exacerbates replication stress and triggers replication catastrophe in HGSC cell lines regardless of their particular driver oncogene

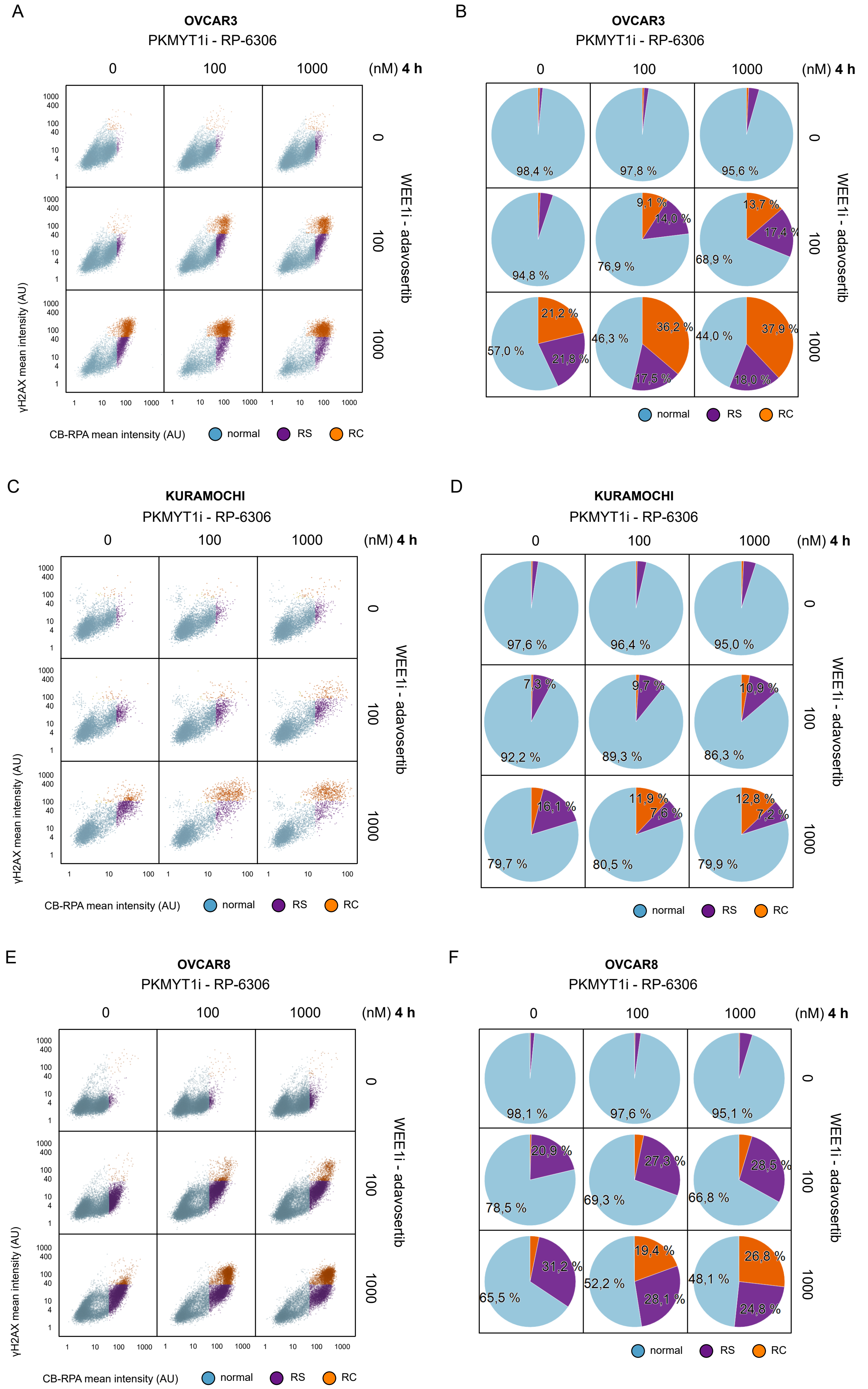

Supplementary Figure 6. - WEE1 and PKMYT1 co-inhibition increases genomic instability and activates cGAS-STING response in OVCAR8 cells

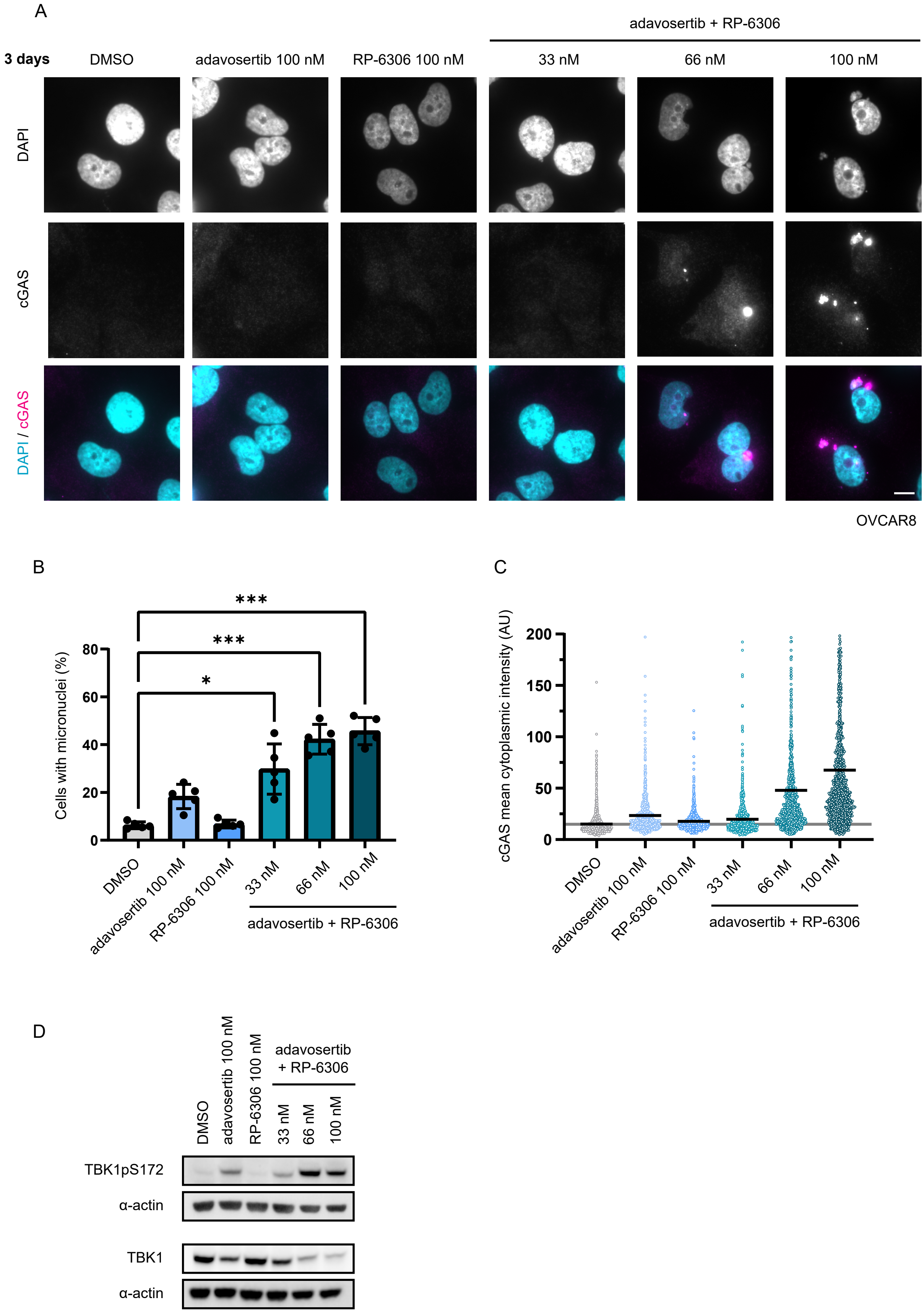

Supplementary Figure 7. - WEE1 and PKMYT1 co-inhibition kills patient-derived ovarian cancer organoids in combination with standard gemcitabine chemotherapy

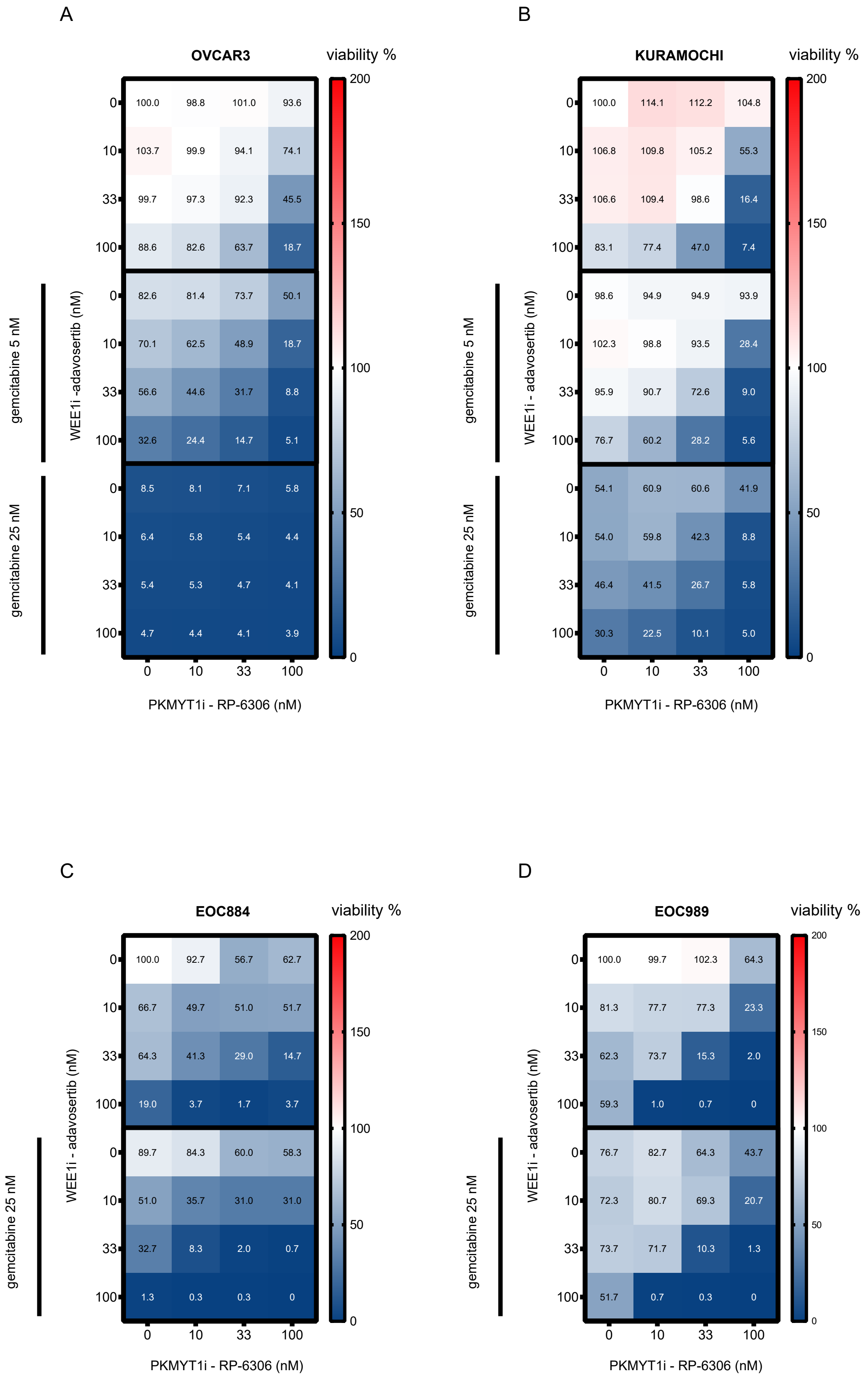

Supplementary Figures

Supplementary Figure 1. - Combined inhibition of WEE1 and PKMYT1 synergize in killing of cancer cells

(A) Dose response matrix for cell viability upon 5-day treatment with WEE1i inhibitor ZN-c3 in combination with RP-6306 in U2OS cells, data represent mean from triplicate. (B) Synergy ZIP scores corresponding to data in (A) presented a synergy landscape. A score ≥ 10 represent synergy, a score ≤ -10 represents antagonism. (C) Western blot analysis of CDK1pY15 and CDK1pT14 upon 4 h treatment either with adavosertib or RP-6306 or in combination in U2OS cells, n = 3. (D) Western blot analysis of CDK1pY15 and CDK1pT14 upon 4 h treatment either with WEE1i inhibitor ZN-c3, RP-6306, or in combination in U2OS cells, n = 3 (E) Proliferation of BJ fibroblast HRAS(G12V) Tet ON without or with doxycycline addition for 48 h, bars indicate mean and SD, n = 25, ****: $P < 0.0001$ (unpaired Student's t test).

Supplementary Figure 2. - WEE1i and PKMYT1i multiple low dose application is tolerated in mouse model

(A) Total plasma levels of RP-6306 and adavosertib in NGX mice treated with RP-6306 (5 mg/kg) adavosertib (15 mg/kg), the combination thereof, or vehicle. Drugs were administered orally twice daily for 21 days at an intermittent dosing schedule., n = 6, bars indicate mean and SD (B) Free plasma levels (corrected for plasma protein binding) of RP-6306 and adavosertib, n = 6, bars indicate mean and SD. (C) The average total and free plasma levels of the 2 timepoints presented in (A) nad (B), Kruskal-Wallis test. (D) Changes in body weight in NGX mice treated as described in (A). Results are expressed as mean body mass ± SD, n = 6 for each cohort. (E) Liver weight normalized to body weight at the end of the treatment described in (A). **: $P \leq 0.01$, Kruskal-Wallis test.

Supplementary Figure 3. - WEE1 and PKMYT1 co-inhibition exacerbates replication stress and triggers replication catastrophe

(A) Representative images related to QIBC analysis of replication stress upon 4 h treatment with adavosertib in combination with RP-6306 in U2OS cells presented in Fig.2A and B, scale bar represent 20 µm (B) Dose response matrix for QIBC analysis of replication stress upon 4 h treatment with WEE1i inhibitor ZN-c3 in combination with RP-6306 in U2OS cells. AU = arbitrary unit, RS = replication stress, RC = replication catastrophe (C) Analysis of relative cell populations percentage from (A) RS = replication stress, RC = replication catastrophe. (D) Representative images related to QIBC analysis of replication stress upon 4 h treatment with ZN-c3 in combination with RP-6306 in U2OS cells presented in Suppl.Fig.3B and C, scale bar represent 20 µm.

Supplementary Figure 4. - WEE1 and PKMYT1 gene expression in ovarian cancer and gene expression of driver oncogenes in panel of HGSC cell lines

(A) Analysis of WEE1 and PKMYT gene expression in ovarian tumor compared to normal ovarian tissue (TCGA dataset); TPM = transcript per million; two-way analysis of variance (ANOVA), *$0.01 < P \leq 0.05$, **$0.001 < P \leq 0.01$, ***$0.0001 < P \leq 0.001$; Analysis was performed in GEPIA (Gene Expression Profiling Interactive Analysis) - http://gepia.cancer-pku.cn/ (DOI: 10.1093/nar/gkx247). (B) Correlation analysis of WEE1 and PKMYT1 gene expression in ovarian tumors (TCGA dataset); R = Pearson correlation coefficient = 0.17, P-value = 0.00054, indicating no correlation; TPM = transcript per million; Analysis was performed in GEPIA (Gene Expression Profiling Interactive Analysis) - http://gepia.cancer-pku.cn/ (C) Relative expression of selected oncogenes, WEE1 and PKMYT1 for panel of HGSC cell lines.

Supplementary Figure 5. - WEE1 and PKMYT1 co-inhibition exacerbates replication stress and triggers replication catastrophe in HGSC cell lines regardless of their particular driver oncogene

(A) Dose response matrix for QIBC analysis of replication stress with adavosertib in combination with RP-6306 in OVCAR3 cells. AU = arbitrary unit, RS = replication stress, RC = replication catastrophe (B) Analysis of relative cell populations percentage from (A) (C) Dose response matrix for QIBC analysis of replication stress with adavosertib in combination with PKMYT1 inhibitor RP-6306 in KURAMOCHI cells. (D) Analysis of relative cell populations percentage from (C). (E) Dose response matrix for QIBC analysis of replication stress with adavosertib in combination with PKMYT1 inhibitor RP-6306 in OVCAR8 cells. (F) Analysis of relative cell populations percentage from (E).

Supplementary Figure 6. - WEE1 and PKMYT1 co-inhibition increases genomic instability and activates cGAS-STING response in OVCAR8 cells

(A) Representative images of micronuclei formation and cGAS activation upon 3-day treatment with adavosertib in combination with RP-6306 in OVCAR8 cells; scale bar represent 10 μm (B) Quantification of micronuclei formation activation upon 3-day treatment with adavosertib in combination with RP-6306 in OVCAR8 cells; bars indicate mean and SD from biological quintuplicate; two-way analysis of variance (ANOVA), *0.01 < P ≤ 0.05, **0.001 < P ≤ 0.01, ***0.0001 < P ≤ 0.001 (C) Quantification of cytoplasmic cGAS intensity upon 3 day treatment with adavosertib in combination with RP-6306 in OVCAR8 cells, bars indicate mean. (D) Western blot analysis of TBK1 activation (TBK1pS172) upon 72 h treatment with adavosertib in combination with RP-6306 in OVCAR8 cells, n = 2.

Supplementary Figure 7. - WEE1 and PKMYT1 co-inhibition kills patient-derived ovarian cancer organoids in combination with standard gemcitabine chemotherapy

(A) Dose response matrix for OVCAR3 cells viability upon 5-day treatment with WEE1i inhibitor adavosertib in combination with PKMYT1 inhibitor RP-6306 without and with 18 h pre-treatment with 5 nM or 25 nM gemcitabine, respectively; mean, n = 3. (B) Dose response matrix for KURAMOCHI cells viability upon 5-day treatment with WEE1i inhibitor adavosertib in combination with PKMYT1 inhibitor RP-6306 without and with 18 h pre-treatment with 5 nM or 25 nM gemcitabine, respectively; mean, n =3. (C) Dose response matrix for EOC884 organoids viability upon 7-day treatment with WEE1i inhibitor adavosertib in combination with PKMYT1 inhibitor RP-6306 without and with 18 h pre-treatment with 25 nM gemcitabine, data represent percentage of live organoids relative to DMSO-treated control, mean, n = 3 (D) Dose response matrix for EOC989 organoids viability upon 7-day treatment with WEE1i inhibitor adavosertib in combination with PKMYT1 inhibitor RP-6306 without and with 18 h pre-treatment with 25 nM gemcitabine, data represent percentage of live organoids relative to DMSO-treated control, mean, n = 3.
